# A high performance lithium ion capacitor achieved by the integration of a Sn-C anode and a biomass-derived microporous activated carbon cathode

**DOI:** 10.1038/srep40990

**Published:** 2017-02-03

**Authors:** Fei Sun, Jihui Gao, Yuwen Zhu, Xinxin Pi, Lijie Wang, Xin Liu, Yukun Qin

**Affiliations:** 1 School of Energy Science and Engineering, Harbin Institute of Technology, Harbin, 150001, China; 2 School of Energy and Safety Engineering, Tianjin Chengjian University, Tianjin, 300384, China

## Abstract

Hybridizing battery and capacitor materials to construct lithium ion capacitors (LICs) has been regarded as a promising avenue to bridge the gap between high-energy lithium ion batteries and high-power supercapacitors. One of the key difficulties in developing advanced LICs is the imbalance in the power capability and charge storage capacity between anode and cathode. Herein, we design a new LIC system by integrating a rationally designed Sn-C anode with a biomass-derived activated carbon cathode. The Sn-C nanocomposite obtained by a facile confined growth strategy possesses multiple structural merits including well-confined Sn nanoparticles, homogeneous distribution and interconnected carbon framework with ultra-high N doping level, synergically enabling the fabricated anode with high Li storage capacity and excellent rate capability. A new type of biomass-derived activated carbon featuring both high surface area and high carbon purity is also prepared to achieve high capacity for cathode. The assembled LIC (Sn-C//PAC) device delivers high energy densities of 195.7 Wh kg^−1^ and 84.6 Wh kg^−1^ at power densities of 731.25 W kg^−1^ and 24375 W kg^−1^, respectively. This work offers a new strategy for designing high-performance hybrid system by tailoring the nanostructures of Li insertion anode and ion adsorption cathode.

Secondary lithium-ion batteries (LIBs) and supercapacitors (SCs) represent two typical and effective electrochemical energy storage systems which show complementary energy-storage features due to their different charge-storage mechanisms^1^,^2^. For instance, LIBs can achieve the high energy density (130~200 Wh kg^−1^) by utilizing Li insertion and desertion active materials, and have been widely applied in laptop computers, mobile phones and electric vehicles^3^,^4^. However, Li storage mechanism in LIBs commonly results in low power densities (<1000 W kg^−1^) due to the sluggish solid-state ion diffusion and Faradaic reactions in bulk electrodes^3^. Moreover, the destruction and volume expansion of electrode structures upon cyclical lithiation/delithiation generally leads to short cycling lives (<1000 cycles) of LIBs^3^,^5^,^6^. Conversely, SCs, especially electrical double layer capacitors, can deliver high power density (>10 kW kg^−1^) and long cycling lifespan (over 10^5^ cycles) due to the extremely rapid ion adsorption-desorption process (with seconds) onto electrode internal surface, since which the energy densities of SCs are very limited (5~10 Wh kg^−1^)^7^,^8^,^9^. For the aim to achieve high energy density without sacrificing high power-output, an effective strategy is to integrate the positive attributes of secondary batteries and of supercapacitors to construct hybrid capacitor-battery system, also called hybrid lithium ion capacitors (LICs), which have been proposed and demonstrated practicable^2^,^10^,^11^. LICs commonly employ a high-power SC electrode (e.g. activated carbon and porous carbon) as cathode, a high-energy LIB electrode (graphite, Si/C, and metal oxides) as anode and a Li salt containing organic electrolyte^2^,^10^. One of the key difficulties in developing high-performance LICs is the imbalance in the power capability and charge storage capacity between the two electrodes^12^. For the high-capacity Li intercalation anodes, the biggest challenge is to restrain the cycling-induced electrode degradation (volume change, pulverization and inactivation), especially at high rates to match the high power characteristic of cathodes^12^,^13^,^14^,^15^. For the cathode, the much lower capacity (30–35 mAh g^−1^) of carbon based cathodes compared with high-capacity Li-ion storage anodes will lead to the low energy density of LICs based on the capacity equation (1/C_LIC_ = 1/C_cathode_ + 1/C_anode_)^12^,^16^,^17^.

In the aspect of optimizing the combination of anode and cathode, various LICs systems have been reported with incremental performances. Earlier, Amatucci *et al*.^18^. reported a hybrid configuration utilizing activated carbon as cathode and nanostructured Li_4_Ti_5_O_12_ as anode with energy density of >20 Wh kg^−1^. Recently, Li *et al*. reported a Li-ion hybrid supercapacitor with the highest energy density so far by coupling an biomass derived activated carbon cathode and a Si-C nanocomposite anode^2^. The hybrid supercapacitor exhibits the highest energy density so far (257Wh kg^−1^ at 867 W kg^−1^). Furthermore, other LIC systems including AC//B-Si/SiO_2_/C^5^, TiC//PHPNC^12^, 3D graphene//Fe_3_O_4_-graphene^19^, SnO_2_–C//porous carbon^20^, AC//hard carbon^21^,^22^, AC//soft carbon^23^, AC//LTO^24^,^25^, SWNT//V_2_O_5_^26^, porous graphene//Li_4_Ti_5_O_12_/C^27^, as well as AC//graphite^28^ have also been successfully explored. Despite those efforts, there is still much room for the exploration and evaluation of new LIC configurations to circumvent the kinetics and capacity discrepancy between cathode and anode. Definitely, the key to a high performance LIC is to couple both high rate capability anode and high capacity cathode materials. Metallic tin (Sn), which shows a high Li storage theoretical capacity (ca. 992 mAh g^−1^ for Li4.4Sn) and an appropriate low discharge potential versus Li/Li^+^, has been considered as one of the most promising alternative anode materials for next generation LIBs^29^. Meanwhile, biomass-derived activated carbons are recognized as the most promising supercapacitor electrode materials due to their multiple merits including high surface areas, vast resources, controllable microstructure and easy for scale-up.

Herein, we report a new LIC configuration by integrating a rational designed Sn-C nano- composite anode with a biomass-derived activated carbon cathode. The unique Sn-C nanocomposite obtained by a facile confined growth strategy possesses multiple structural merits including well-confined small Sn nanoparticles, homogeneous distribution, interconnected spherical framework with large surface area and nitrogen-rich carbon lattice, synergically enabling the fabricated anode with high Li storage capacity and excellent rate capability. To increase the capacity of the high-rate capacitive cathode, we demonstrate a new type of high surface area activated carbon (PAC) derived from the simple activation of pomelo peel. Coupling these two electrode materials gives a high-performance hybrid LIC (Sn-C//PAC) that can be operated between 2.0~4.5 V and exhibit high energy densities of 195.7 Wh kg^−1^ and 84.6 Wh kg^−1^ at power densities of 731.25 W kg^−1^ and 24375 W kg^−1^, respectively. These results are remarkable compared with the state-of-the-art reports for LICs, and also offers a new platform for designing high-performance hybrid supercapacitors by tailoring the nanostructures of both anode and cathode.

## Results and Discussion

### Sn-C anode construction

The Sn-C nanocomposite was prepared by simple liquid impregnation of a tin salt solution on a well-designed N-rich mesoporous carbon framework, followed by thermal treatment. Small Sn nanoparticles homogeneously confined inside the mesopores of the carbon network are obtained in this way. The nanoconfinement concept has been applied preciously to downsize the metallic particles and to limit particle coalescence during electrochemical process^30^. In our work, by synergically controlling the morphology, pore structure and N-doping level, we firstly prepared a novel kind of N-rich mesoporous carbon (NRMC) spheres as the carbon matrix for Sn nanoparticle confined growth. The formation of NRMC matrix is based on an aerosol-assisted continuous spraying method, as illustrated in Figure S1. More specially, an aqueous precursor solution containing melamine-phenolic-formaldehyde (MPF) resins and colloidal silica template undergo an atomization process, co-polymerization process, carbonization treatment and ultimate template removal process to yield the NRMC. Figure S2 gives the structural properties of NRMC spheres. Such carbon spheres possess high surface area, uniform mesoporous structure, and ultrahigh level of N-doping (14.51%), favorably ensuring the grimly confined growth of Sn nanoparticles in the carbon framework. The as-formed NRMC is impregnated with the corresponding quantity of SnCl_2_ ethanol solution and followed by thermal treatment to obtain the Sn-C nanocomposites.

**Figure 1:**
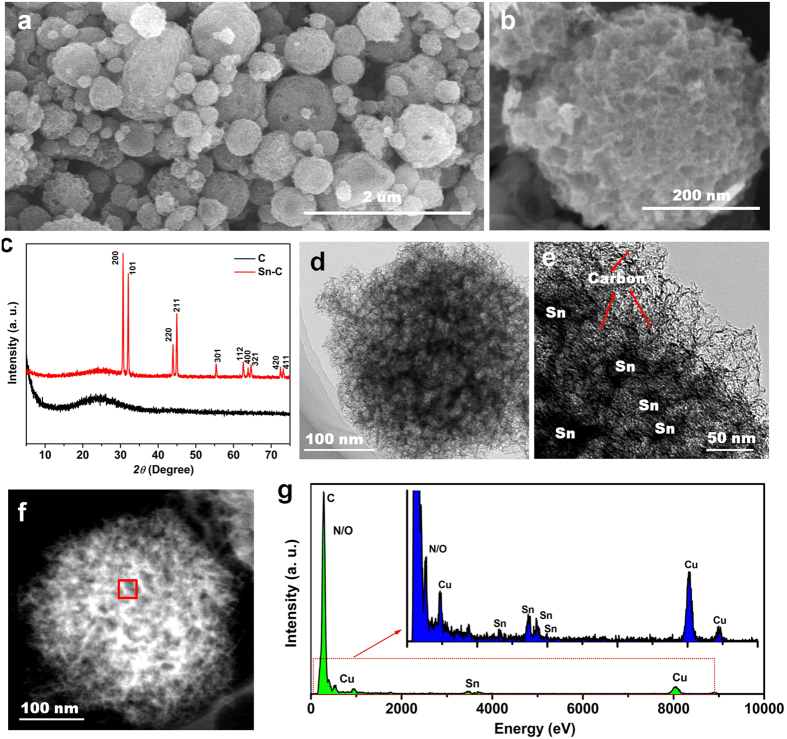
Structural characterization of Sn-C nanocomposite. (**a**) Low-magnification SEM image of Sn-C. (**b**) High-magnification SEM image of ZnO-NMPCS. (**c**) XRD patterns of carbon framework and Sn-C. (**d**) Representative TEM image of a Sn-C nanoparticle. (**e**) HRTEM image of Sn-C. (**f**) HAADF-STEM image of a Sn-C nanoparticle. (**g**) Corresponding EDX spectrum of red box in (**f**).

Figure 1(a) and (b) show scanning electron microscopy (SEM) images of the Sn-C, which exhibit a polydispersed spherical morphology greatly inheriting the spherical architecture of the carbon matrix (shown in Figure S2a). Moreover, there is no substance exposed on outer surface of the carbon spheres (Fig. 1b), which suggests that all the tin salt can be impregnated into internal pores of carbon matrix and give the resulting Sn nanoparticles grimly growing inside the pores of carbon framework. Figure 1(c) show the X-ray diffraction (XRD) patterns of Sn-C composite and pure carbon matrix. Obviously, the N-rich mesoporous carbon exhibits an amorphous nature. All the diffraction peaks of Sn-C composite can be readily indexed to metal Sn (JCPDS card No. 04-0673)^29^,^31^. Figure 1(d) and (e) shows the representative transmission electron microscopy (TEM) images of Sn-C nanocomposite, further confirming that Sn nanoparticles (black dots) are homogeneously confined inside the carbon (grey color) matrix. Figure 1(f) and (g) illustrates the high-angle annular dark-filed scanning TEM (HAADF-STM) images and corresponding EDX analysis results, undoubtedly demonstrating the presence of C, O, N and Sn in the Sn-C composite. The Cu signal observed in the EDX image is due to the Cu substrate for TEM test. Furthermore, Figure S3 shows the thermogravimetry analysis (TGA) result of Sn-C in air, evidencing that the Sn content in the composite is ~40 wt% by corresponding the resultant SnO_2_ to Sn. This mass loading of Sn has shown to be the optimal for assuring the homogeneous distribution. Figure S4 further illustrates the large-scale SEM image and elemental distribution of the Sn-C composite, revealing the highly dispersed Sn in the carbon matrix with approximate 40% weigh percentage.

**Figure 2:**
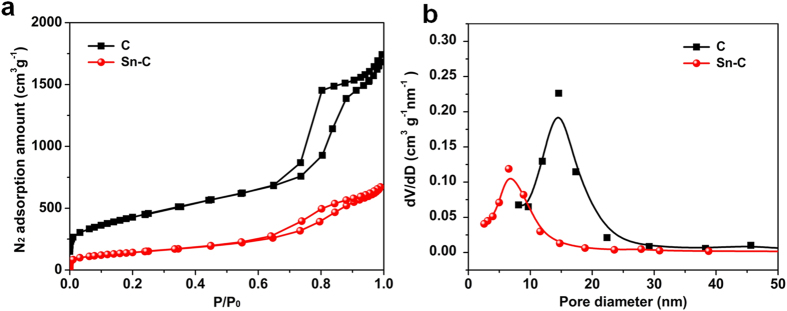
(**a**) Nitrogen sorption isotherm of carbon and Sn-C. (**b**) BJH method based pore size distribution of carbon and Sn-C.

Figure 2(a) and (b) show the N_2_ adsorption isotherms and the corresponding pore size distributions of Sn-C and C. Both typical IV isotherms with a hysteresis loop within a relative pressure P/Po of 0.6-1 indicate a mesoporous structure^32^. The pore size of carbon mainly distributes between 10 and 20 nm which is consistent with the particle size of employed silica template. The highly mesoporous structure of carbon yields a high surface area up to 1560 m^2 ^g^−1^ and high pore volume up to 2.56 cm^3 ^g^−1^. Such uniform mesopores, large surface area and high pore volume could ensure the efficient diffusion path and abundant accommodation space for Sn species diffusion and growth. Notably, after Sn species confined growth, Sn-C shows both decreased N_2_ sorption amount and pore size due to the Sn embedding, but still maintains a considerable surface area of 389 m^2^ g^−1^ and pore volume of 0.84 cm^3^ g^−1^. During discharge/charge process, the mesoporous structure of Sn-C facilitates the diffusion of lithium ions through the electrode, alleviates the expansion of Sn-species and relieves the pulverization of electrodes.

**Figure 3:**
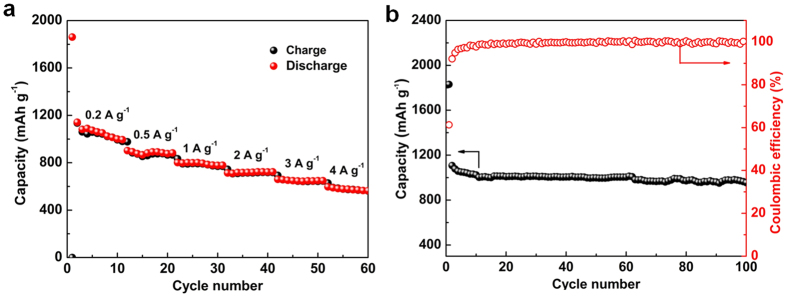
Electrochemical performances of Sn-C. (**a**) Rate performances at various current densities from 0.2 A g^−1^ to 4 A g^−1^. (**b**) Cycling performances at a current density of 0.2 A g^−1^.

All of aforementioned results demonstrate that the as-prepared Sn-C composite encompasses four key merits as high performance anode materials for LIBs, namely, small confined Sn nanoparticles, homogeneous distribution within carbon framework, interconnected mesoporous structure with high surface area and also high nitrogen doing level. To evaluate the Li storage capability of Sn-C, coin cells (2032) with a metallic Li counter electrode were constructed and tested. Figure 3(a) shows the representative electrochemical performance of Sn-C anodes in a voltage range of 0.01~3 V (*vs*. Li/Li^+^). As shown in Fig. 3(a), Sn-C exhibits excellent rate performances. Increasing the current densities from 0.2 to 4 A g^−1^, the reversible capacities are ca. 1081, 995, 877, 777, 721, and 649 mAh g^−1^ at 0.2, 0.5, 1, 2, 3, and 4 Ag^−1^, respectively. Moreover, the Sn-C also shows outstanding cycling performance in Fig. 3(b), from which Sn-C can maintain a high Li storage capacity of 995 mAh g^−1^ after 100 charge-discharge cycles at 0.2 A g^−1^. It is also worth noting that the Coulombic efficiency of Sn-C remains around 99.7% during long-time cycling. Such excellent rate performance and Li storage capacity are also among the highest levels of reported Sn based anode^29^,^33^,^34^,^35^,^36^,^37^,^38^,^39^. Figure S5 displays the impendence spectra of fresh Sn-C electrode and Sn-C electrode after 100 cycles at 0.2 A g^−1^. The fitting resistances of each component in Sn-C before and after cycling are also calculated. The ohmic resistance (Rs) and charge transfer resistance (RCT) of fresh Sn-C anode are 6.5 and 55.5 Ohms. After cycling, Sn-C electrode still maintains an acceptable impendence characteristic with a lower Rs of 5.8 Ohm and a slightly higher RCT of 65.5 Ohm. Moreover, the cycled Sn-C anode displays a decreased slope in the low-frequency region, suggesting the deteriorative Li-ion diffusion. Based on the structural characterization, such excellent anode performance of our Sn-C nanocomposite must stem from the confined growth of Sn nanoparticles within mesoporous carbon framework, which not only alleviate the inherent shortcomings including volume change and particle aggregation facing Sn metal anode, but also ensure the sufficient Li^+^ storage space/sites and fast ions/electrons transport, thus demonstrating excellent lithium storage properties. Hereto, we have demonstrated the superior anode performance of our Sn-C composite that should be a desirable anode choice for Li ion hybrid supercapacitors.

### Biomass-derived activated carbon cathode

**Figure 4:**
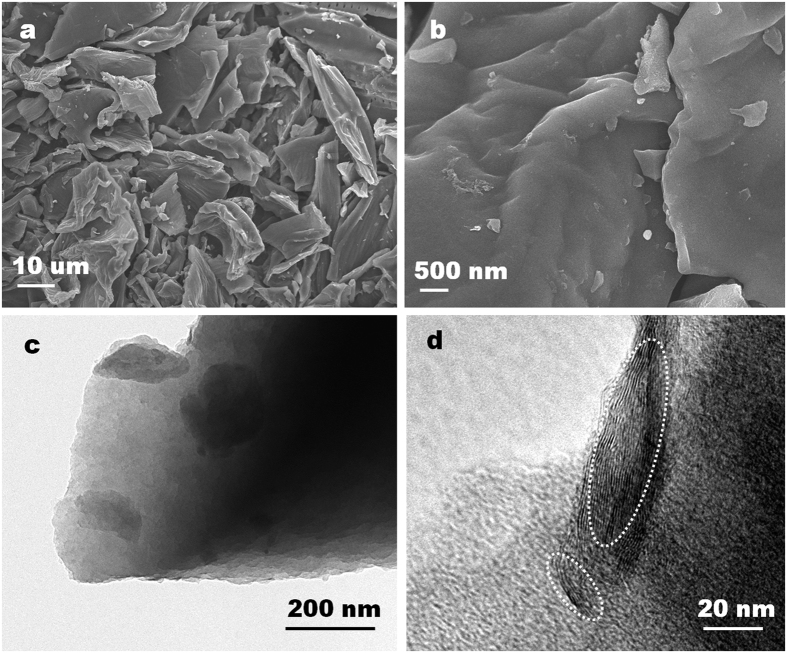
Representative SEM and TEM images of PAC-900. (**a**) Low-magnification SEM image of PAC-900. (**b**) High-magnification SEM image of PAC-900. (**c**) Low-magnification TEM image of PAC-900. (**d**) HRTEM image of PAC-900.

To boost the energy density of the constructed hybrid capacitor, a high-performance anion adsorption cathode is indispensable. In this work, we prepare microporous activated carbon derived from biomass waste pomelo peel by a simple KOH activation process. The obtained pomelo peel derived carbon (PAC) was paired with the Sn-C anode for the construction of LICs, demonstrating outstanding performance. Figure 4 shows the SEM and TEM images of optimized PAC (PAC-900) under 900 °C. As shown in Fig. 4(a) and (b), the PAC-900 mainly consists of aggregated small and smooth carbon sheets with size of tens of microns. The TEM image (Fig. 4c) of PAC-900 further demonstrates the sheet structure of PAC-900 which is similar to the stacking graphene structure. Figure 4(d) shows the HRTEM of PAC-900, conforming a mainly amorphous structure accompanied by evident multilayer graphene structure (within the white oval shapes). Comparably, no graphene or graphite lattice fringe can be observed for the PAC-700 and PAC-800, which ascribes to the lower activation temperature^2^ (Figure S6). X-ray diffraction (XRD) studies also indicate the mainly amorphous structure for PACs (Figure S7).

**Figure 5:**
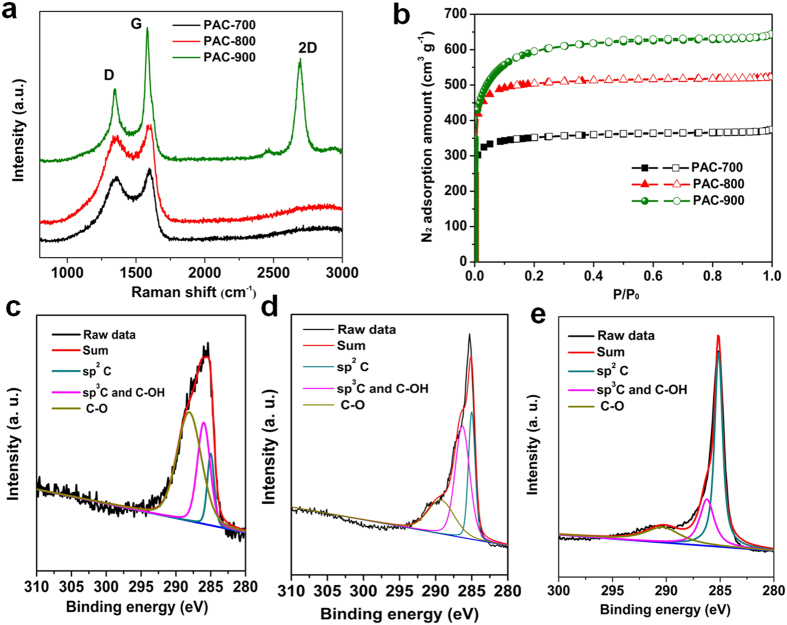
(**a**) Raman spectra of PACs. (**b**) N_2_ adsorption isotherms of PACs. (**c**) C1s spectrum of PAC-700. (**d**) C1s spectrum of PAC-800. (**e**) C1s spectrum of PAC-900.

Figure 5(a) shows the Raman spectra of the PACs, of which the peaks at ~1350 and ~1600 cm^−1^ represent the D and G band of the carbon framework structure, respectively^40^. The intensity ratio of *I*_*G*_ and *I*_*D*_ depends on the graphitization degree^2^. As shown in Fig. 5(a), elevating the activation temperature could result in increasing *I*_*G*_/*I*_*D*_ ratios. Notably, when the activation temperature reached 900 °C, the *I*_*G*_/*I*_*D*_ increased sharply to ~2.4, suggesting a high graphitization level. Interestingly, the Raman spectrum of PAC-900 also exhibit obvious 2D band at ~2700 cm^−1^ which is the characteristic peak of several-layer graphene structure^41^, according well with the HRTEM result in Fig. 4(d). The high graphitized structure of PAC-900 may effectively enhance the electron conductivity of fabricated electrode and the rate performance thereof refs 2,11. Figure 5(b) shows the N_2_ adsorption/desorption isotherms of all the PAC samples, which exhibit a typical Type I adsorption isotherms, suggesting the dominant microporous structure. The detailed information about the microstructure characteristic of PAC samples is shown in Table S1. As the activation temperature increasing, the N_2_ adsorption amount greatly increases with PAC-900 possessing the highest Brunauer-Emmett-Teller (BET) surface area and pore volume of 2167 m^2^ g^−1^ and 0.98 cm^3^ g^−1^, respectively. Such high surface area will help in delivering higher capacitance in LICs^2^,^12^.

X-ray photoelectron spectroscopy (XPS) analysis was conducted to investigate the chemical environment of PACs. The XPS based element contents of PACs are summarized in Table S1, from which, as the activated temperature increases, the oxygen content decreases sharply from 33.69 at-% for PAC-700 to 1.77 at-% for PAC-900. As well known, the oxygen-containing functional groups in carbon materials could significantly affect the capacity and cycling life for electrochemical process^2^. The high-resolution C1s XPS spectra of PACs (Fig. 5c–e) can be deconvoluted into mainly three peaks representing *sp2* carbon (245 ± 0.2 eV), *sp3* carbon or C-OH (286 ± 0.2 eV), C-O (287 ± 0.2 eV)^11^. It can be seen that compared with PAC-700 and PAC-800, PAC-900 possesses the highest percentage of *sp*^2^ carbon component and lower ratio of oxygen-containing groups. The high carbon purity of PAC-900 is considered to enhance the cycling stability of fabricated electrodes^2^.

**Figure 6:**
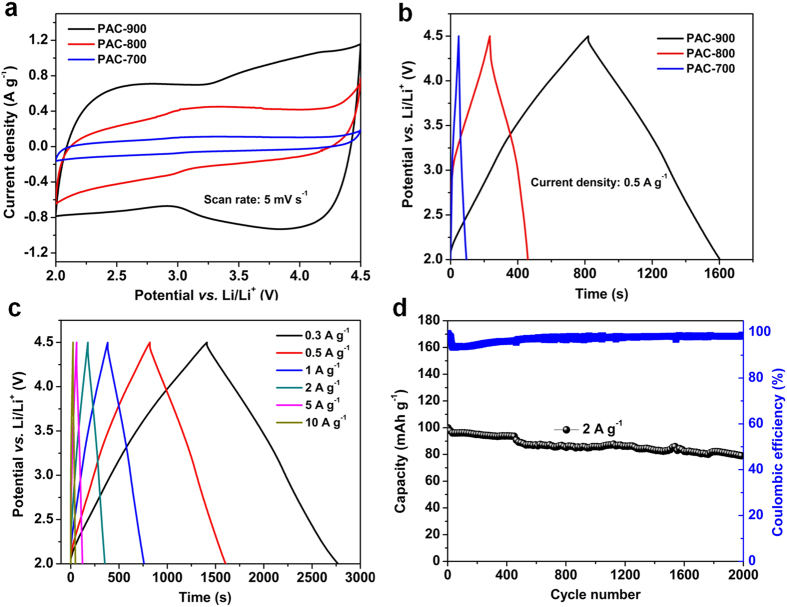
Electrochemical performance of PACs in half cell configurations. (**a**) Cyclic voltammograms (CVs) of PACs within the range of 2.0~4.5 V at a scan rate of 5 mV s^−1^ (**b**) Galvanostatic charge-discharge curves of PACs at a current density of 0.5 A g^−1^. (**c**) Galvanostatic charge-discharge curves of PAC-900 at various current densities. (**d**) Cycling performance of PAC-900 at 2 A g^−1^.

The electrochemical performances of PACs were comparably evaluated by half-cell configurations using cyclic voltammetry (CV) and galvanostatic charge-discharge (GC) process. Figure 6(a) shows the CV profiles of PACs at a scan rate of 5 mV s^−1^ in the voltage window of 2.0~4.5 V, which show quasi-rectangular shapes indicating the capacitive behavior^5^,^11^,^12^. Compared with PAC-900, PAC-800 and PAC-700 show obviously inferior capacitances, which can also be supported by the GC curves in Fig. 6(b). Based on the linear charge/discharge profiles at 0.5 Ag^−1^, the specific capacitance of PACs based electrodes are 6.57 mAh g^−1^ (9.46 F g^−1^), 31.9 mAh g^−1^ (46 F g^−1^) and 112 mAh g^−1^ (161 F g^−1^) for PAC-700, PAC-800 and PAC-900 respectively. Figure 6(c) further illustrate the rate performance of PAC-900 at the current densities of 0.3 A to 10 Ag^−1^. At a low current density of 0.3 A g^−1^, PAC-900 exhibit a specific capacity of 115 mAh g^−1^ (166 F g^−1^). Even at a high current density of 10 A g^−1^, the PAC-900 still provide a gravimetric capacitance of 100 F g^−1^ (70 mAh g^−1^), which is superior to the performance of reported activated carbon cathodes, especially at high rates^2^. Considering the aforementioned structure merits of PAC-900, such high capacity and rate performance should be ascribed to the synergy of high surface area with microporosity, high degree of graphitization, and high carbon purity with low heteroatom contents. Furthermore, PAC-900 also exhibit excellent cycling performance with ~80% capacity retention after 2000 cycles at a relatively high current density of 2 A g^−1^ (Fig. 6d).

### Sn-C//PAC hybrid capacitor construction

**Figure 7:**
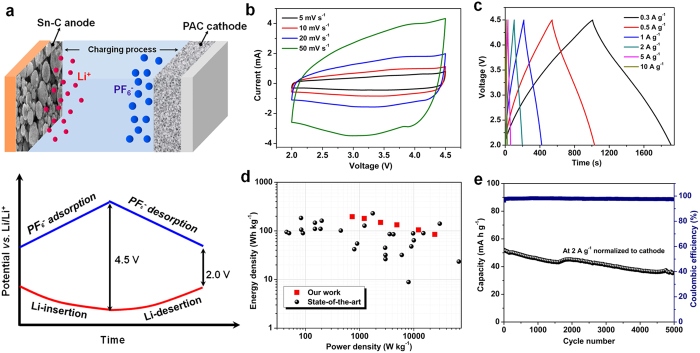
Mechanism and electrochemical performances of Sn-C//PAC. (**a**) Schematic illustration of work mechanism of Sn-C//PAC. (**b**) Representative cyclic voltammograms (CVs) within the range of 2.0~4.5 V at various scan rates. (**c**) Galvanostatic charge-discharge curves at various current densities. (**d**) Ragone plot in comparison with literature. (**e**) Cycling performance at 2 A g^−1^.

The LIC full cells are assembled using pre-lithidated Sn-C nanocomposite as the anode materials and fresh PAC-900 as the cathode materials (the hybrid supercapacitor denoted as Sn-C//PAC). After optimization, the mass ratio of Sn-C and PAC-900 is fixed as 1:3 to obtain the best energy/power density. Figure 7(a) shows a detailed work mechanism of Sn-C//PAC in the voltage range of 2.0~4.5 V. During the charge process, PF6^−^ ions are accumulated on the interface of PAC electrode/electrolyte while Li^+^ ions insert into Sn-C anode by the formation of LixSn alloy, probably accompanied with the Li adsorption mechanism of carbon matrix. In the discharging process, PF6^−^ ions and Li^+^ flee from the PAC cathode and Sn-C anode respectively. Figure 7(b–d) show the electrochemical performances of Sn-C//PAC. The CV curves of Sn-C//PAC shown in Fig. 7b exhibit quasi-rectangular shapes and with increasing the scan rate from 5 mV s^−1^ to 50 mV s^−1^, the CV shapes show hardly distortion, indicating an excellent rate performance. Figure 7(c) shows the voltage profiles at different current densities, revealing a little deviation from the linear slope which should stem from the overlapping effects of two different energy-storage mechanisms in cathode (non Faradaic reaction) and anode (Faradaic reaction)^2^,^11^,^12^.

Figure S8 shows the Nyquist plot of the Sn-C//PAC hybrid capacitor which displays similar resistance characteristics to the Sn-C half cell. The magnitude of fitted Rs and RCT are 5.9 and 59.5 Ω respectively, revealing a relatively low resistance in the organic electrolyte. The energy and power densities of Sn-C//CAC hybrid supercapacitor can be calculated based on the galvanostatic charge/discharge curves and the resulting Ragone plot (based on the total mass of the active materials on two electrodes) is shown in Fig. 7(d). It can be observed that our Sn-C//CAC LIC exhibits a high energy density of 195.7 Wh kg^−1^ at 731 W kg^−1^ while maintains 84.6 Wh kg^−1^ with an high power density of 24375 W kg^−1^. In addition, the Sn-C//PAC system also shows an excellent cycling stability of 70% capacity retention in 5000 cycles at a high current density of 2 A g^−1^ (Fig. 7e). Various LIC hybrid systems in previous studies are also compared in Fig. 7(d), from which the energy and power properties of our LIC system are among the highest levels in previous literature such as such as LTO//AC system, MnO//AC system, SnO_2_–C//porous carbon system, Fe_3_O_4_/G//3DGraphene system, AC//soft carbon system, SWNT//V_2_O_5_ system and B-Si/SiO_2_/C//AC system. A comprehensive summarization table which contains most reported advanced Li ion hybrid systems^5^,^11^,^12^,^19^,^20^,^23^,^42^,^43^,^44^,^45^,^46^,^47^,^48^,^49^,^50^,^51^,^52^,^53^,^54^,^55^,^56^,^57^ is also provided as Table S2 in the Supporting Information. Based on the total mass of hybrid supercapacitor^58^, including Cu and Al foil, electrolyte, separator and active materials (a weight ratio of 15.1% for the active materials in a packaged device), the LIC device can deliver maximum energy and power density of 29.6 Wh kg^−1^ and 3680 W kg^−1^, respectively.

## Conclusions

In summary, a new LIC system was designed by coupling a rational designed Sn-C composite anode and a novel activated carbon cathode. The Sn-C nanocomposite was prepared based on a nano-confinement process through rationally designing the N-rich mesoporous carbon matrix and subsequent liquid impregnation. The as-obtained Sn-C composite integrate many structural merits for electrochemical process, including well-confined small Sn nanoparticles, homogeneous distribution, interconnected spherical framework with large surface area and nitrogen-rich carbon lattice which synergically enable the fabricated anode with high Li storage capacity and excellent rate capability. The activated carbon features large surface area, highly microporosity, high carbon purity and high graphitization level, enabling the cathode with a high capacity of as high as 115 mAh g^−1^ (167F g^−1^), as well as excellent rate capability and cycling performance. The hybrids Li-ion capacitor (Sn-C//PAC) was then assembled using the PAC as the cathode materials and Sn-C nanocomposites as the anode material and characterized in the voltage window of 2.0~4.5 V. The Sn-C//PAC exhibits high energy densities of 196~85 Wh kg^−1^ at power density from 731 to 24375 W kg^−1^, as well as good cycling stability with 70% retention after 5000 cycles. These results are remarkable compared with the state-of-the-art reports for LICs systems. This work also provides insights into designing high-performance hybrid supercapacitors by tailoring the nanostructures of both anode and cathode.

## Methods

### Preparation of Sn-C nanocomposite

The carbon matrix for Sn-C nanocomposite formation was prepared by a aerosol-assisted spraying process. Typically, pre-polymerized precursor solution containing 5.044 g of melamine, 2.5 g of phenol, 20 mL formalin aqueous solution (37 wt%) and 25 g colloidal silica solution (30-wt%, SNOWTEX-ST-O, Nissan Chemicals. Inc.) was sent through an atomizer using nitrogen as a carrier gas to form continuously aerosol droplets, which were subsequently passed through a heating furnace with the temperature of 450 °C. The products further experienced a 900 °C carbonization process and washing process (10 wt% HF and de-ionized water) to yield the N-rich mesoporous carbon matrix. For the synthesis of Sn-C nanocomposite, 60 mg N-rich mesoporous carbon was placed in a vial. An aqueous solution containing 50 mg SnCl_2_ dissolving in 5 mL ethanol was added drop by drop until the Sn content attains about 40 wt% with respect to the quantity of carbon in the composite. The mixture was agitated under vacuum condition until complete evaporation of ethanol. Afterwards, the product was thermally treated in a tubular furnace firstly at 280 °C for 5 h and then at 700 °C for 5 h for 3 h with a heating rate of 8 °C min^−1^ under Ar atmosphere. The obtained material was denoted Sn-C.

### Preparation of microporous activated carbon

For the preparation of pomelo peel derived activated carbon (PAC), fresh pomelo peel was firstly washed, drying at 80 °C for 12 h and then cut and grinded into solid powder. The treated pomelo peel experienced the pre-carbonization (700 °C for 1 h under N_2_ flow) and subsequently KOH activation (mass ratio of 1:4) process to obtain the PAC samples. PAC samples under activation temperatures of 700, 800 and 900 °C were prepared and characterized. Accordingly, the PAC samples were denoted PAC-700, PAC-800, PAC-900 respectively.

### Materials Characterizations

Scanning electron microscopy (JSM- 7401 F) and transmission electron microscopy (TEM, JEOL-2010) were used to investigate the morphology and microstructures of prepared samples. The EDS element distribution of Sn-C composite was investigated by high-angle annular dark-filed scanning TEM (HAADF-STM). Pore structure was determined by N_2_ adsorption at −196 °C using ASAP 2020 volumetric sorption analyzer. The pore size distributions were obtained from the adsorption branch of isotherm using the Barrett-Joyner-Halenda (BJH) model. The X-ray diffraction (XRD) measurements were examined on a Rigaku D/Max 2400 diffractometer by using CuKa radiation (40 kV, 100 mA, λ = 1.5406 Ǻ). X-ray photoelectron spectroscopy (XPS) analysis of N-rich carbon framework and PACs were performed on a PHI 5700 ESCA system using AlKa X-ray at 14 kV and 6 mA. Raman spectroscopy was examined on a Renishaw inVia Micro-Raman spectrometer at 532 nm. Thermogravimetric analysis (TGA) of Sn-C was carried out using a thermogravimetric analyzer (TA instruments) with a heating rate of 8 °C min^−1^ in air.

### Electrochemical measurements

Sn-C and PAC samples were firstly examined as anode and cathode materials using half cell configurations with Li metal foil as the counter electrode. For fabrication of Sn-C anode, a typical slurry method was used as followed. Sn-C active materials, carbon black and polyvinylidene fluoride (PVDF) binder with a mass ratio of 7:1.5:1.5 dissolving in N-methyl-2-pyrroli-dinone (NMP). After coating the above slurries on Cu foils, the electrodes were dried at 80 °C under vacuum for 12 h with ~1.0 mg cm^−2^ mass loading of active material. PAC-based cathodes were prepared by homogeneously casting the slurry consisting of 80 wt% of the active material, 10 wt% of carbon black, and 10 wt% of PVDF on carbon-coated aluminum foil with ~3 mg cm^−2^ mass loading of active material. The electrolyte used in the coin cells was 1 M LiPF_6_ in ethylene carbonate and dimethyl carbonate (EC:DEC = 1:1) to obtain the optimal capacity and ion conductivity. Glass fiber (GF/D) was used as separator. The Sn-C//PAC hybrid supercapacitor was fabricated by coupling a pre-lithiated Sn-C anode (pre-cycled 3 times, ending in a lithiated state at ~0.5 V) and a fresh PAC electrode. The mass ratio of PAC to Sn-C is approximately 3:1. All the coin cells were assembled in the glove box with oxygen content less than 1 ppm.

Galvanostatic charge-discharge tests were performed on a Land CT20001A (China) and CV curves were measured by Bio-Logic-VMP3 workstation. The voltage window for the Sn-C electrode and the PAC electrode were 0.01~3 V and 2.0~4.5 V versus Li/Li^+^, respectively. EIS study was performed using Bio-Logic-VMP3 workstation in a frequency range of 100 kHz to 10 MHz at an amplitude of 10 mV. The specific capacitance (F g^−1^) was calculated by the equation listed below: F g^−1^ = mAh g^−1^ • 3.6/ΔV, where ΔV is the voltage window. For Sn-C//PAC hybrid supercapacitor test, the voltage window was set as 2.0~4.5 V; the adopted current density was normalized to the mass of active materials on cathode; the material level energy density and power density of Sn-C//PAC were calculated according to: P = U • I/m; E = P • Δt. Where U = (U_max_ + U_min_)/2, U_max_ and U_min_ are respectively the potential at beginning of discharge and at the end of discharge; I is the constant current; Δt is the discharge time; m is the total mass of active materials on both the cathode and the anode.

## Additional Information

**How to cite this article:** Sun, F. *et al*. A high performance lithium ion capacitor achieved by the integration of a Sn-C anode and a biomass-derived microporous activated carbon cathode. *Sci. Rep.* **7**, 40990; doi: 10.1038/srep40990 (2017).

**Publisher's note:** Springer Nature remains neutral with regard to jurisdictional claims in published maps and institutional affiliations.

## Supplementary Material

Supporting Information
