# Supplementary material for: A high performance lithium ion capacitor achieved by the integration of a Sn-C anode and a biomass-derived microporous activated carbon cathode
Source: Sci Rep. 2017 Feb 3;7:40990. doi: 10.1038/srep40990 (PMC5290747; doi:10.1038/srep40990)
Supplement: Supporting Information [file srep40990-s1.pdf]

Supporting Information for

A high performance lithium ion capacitor achieved by the integration of a Sn-C anode and a biomass-derived microporous activated carbon cathode

Fei Sun[1], Jihui Gao[1]*, Yuwen Zhu[1,2], Xinxin Pi[1], Lijie Wang[1], Xin Liu[1] and Yukun Qin[1]

[1]School of Energy Science and Engineering, Harbin Institute of Technology, Harbin, 150001, China.

[2]School of Energy and Safety Engineering, Tianjin Chengjian University, Tianjin, 300384, China

*To whom correspondence should be addressed. Email: J. Gao (gaojh@hit.edu.cn).

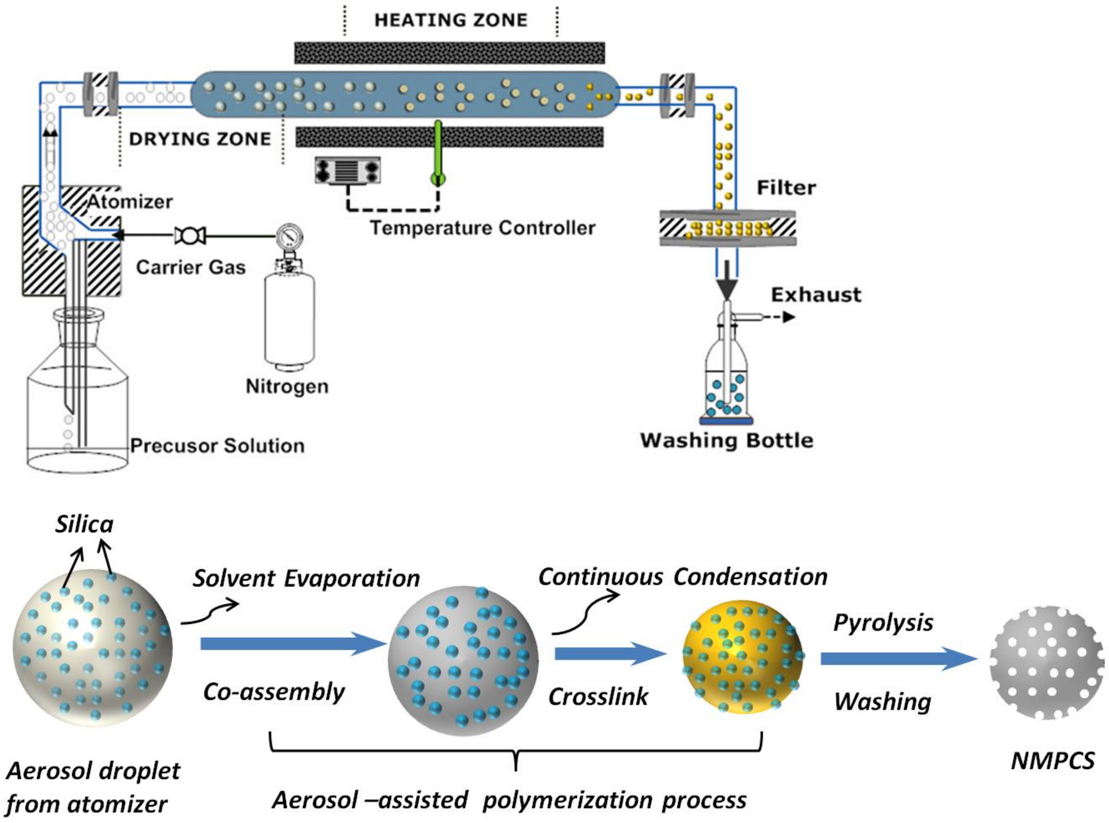

Figure S1. Schematic illustration of the formation of N-rich mesoporous carbon spheres. Aqueous precursor solution containing melamine-phenolic-formaldehyde (MPF) resins and colloidal silica template (with silica particle size in the range of 10~15 nm) undergo an atomization process with nitrogen carrier gas to form continuously generated aerosol droplets, which then pass through a heating oven and convert into copolymer-silica nanocomposites. Subsequent carbonization and further removal of the silica templates result in the carbon spheres with well-designed mesoporous structure and ultrahigh nitrogen content. Particularly worth mentioning is that preparation of carbon spheres by aerosol-assisted continuous spraying process provides an attractive path for structure-controllable synthesizing and can be easy to scale up.

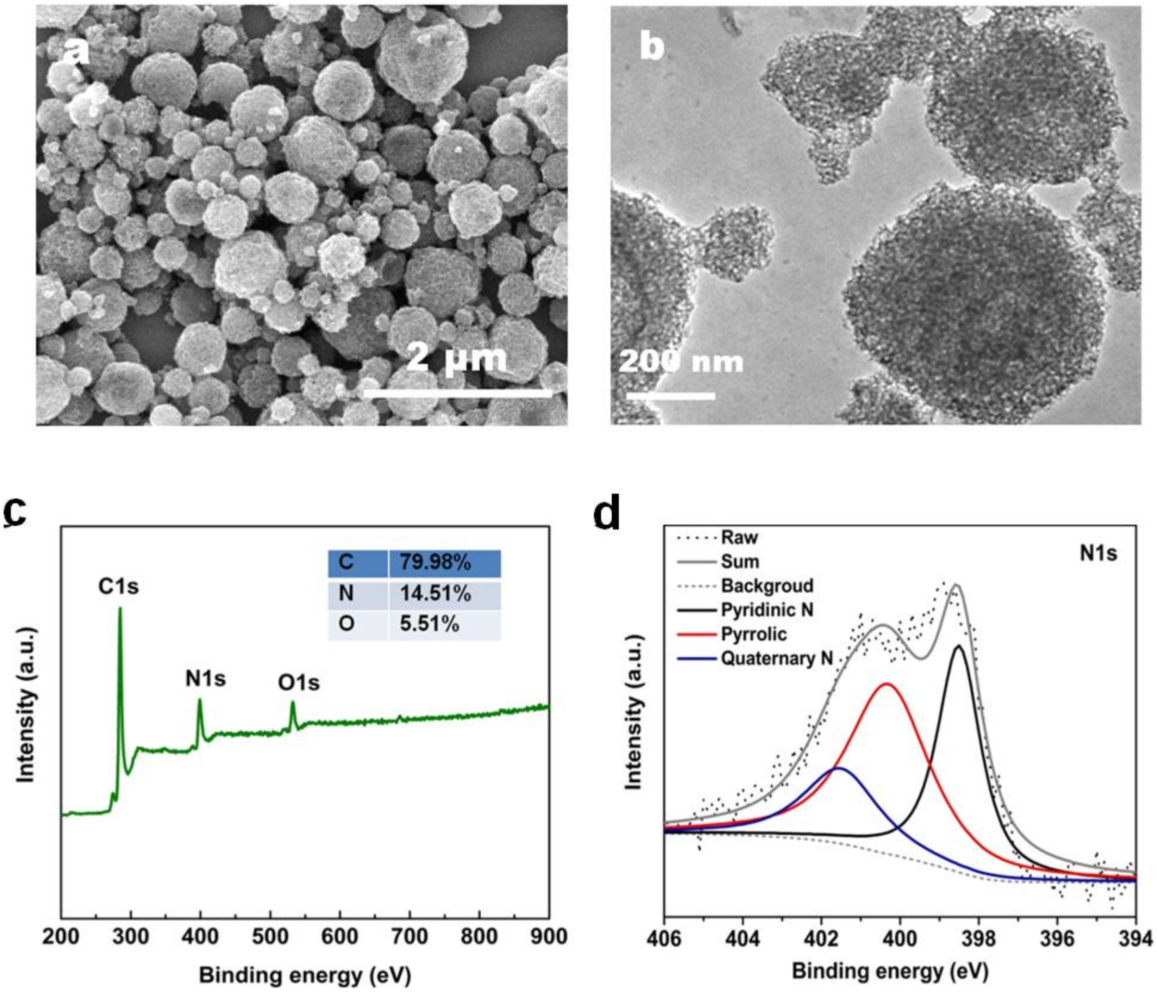

Figure S2. (a) Representative SEM image of N-rich mesoporous carbon. (b) Representative TEM image of N-rich mesoporous carbon. (c) XPS survey spectrum of N-rich mesoporous carbon. (d) N1s spectrum of N-rich mesoporous carbon.

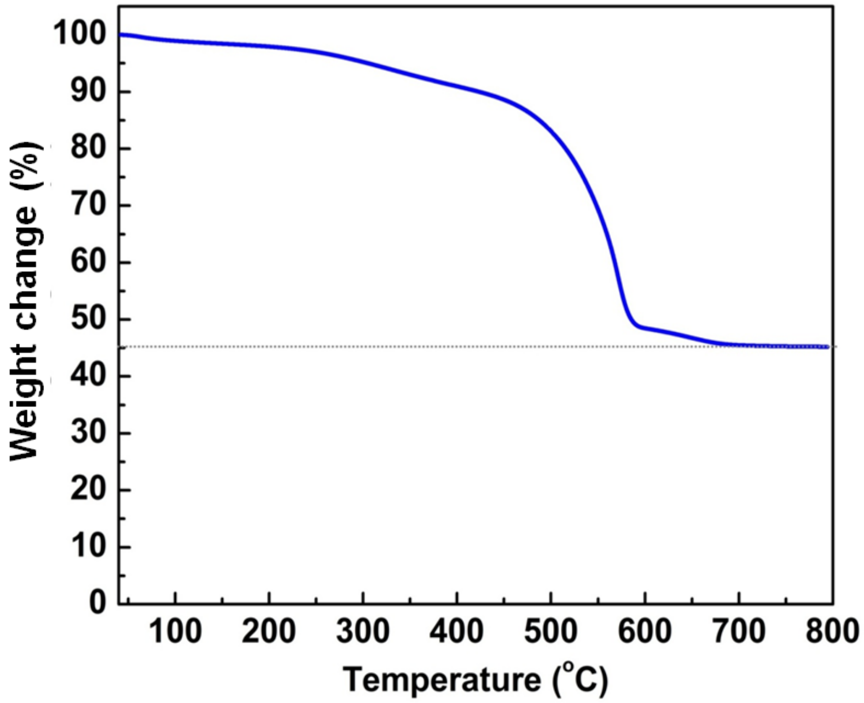

Figure S3. TGA curves of Sn-C in air atmosphere.

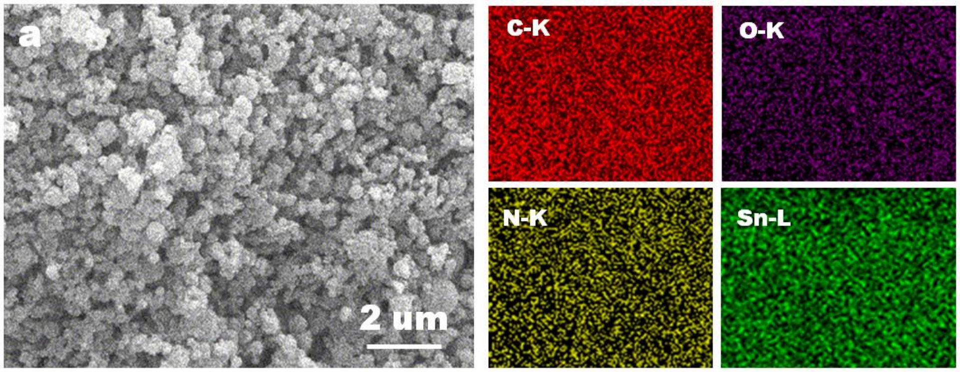

Element	*Wt%*	*At%*
CK	42.38	46.03
NK	11.52	10.58
SnL	43.34	40.78
OK	2.24	2.61

Figure S4. SEM image and EDX elemental distribution of Sn-C composite

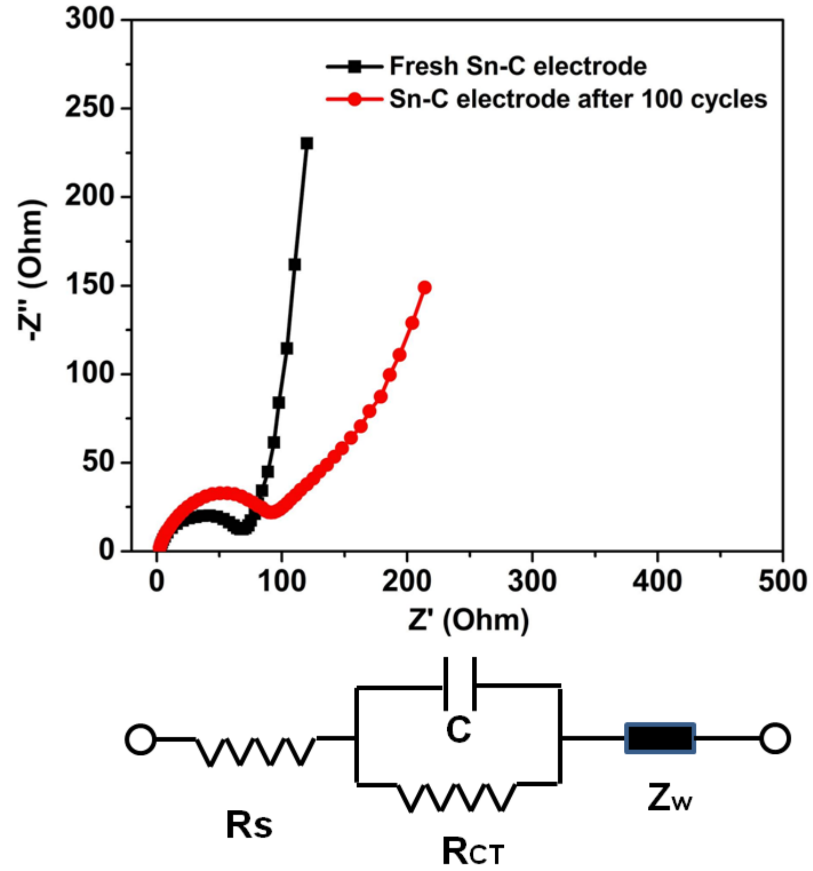

Figure S5. Nyquist plots and fitting circuit of Sn-C anodes in half cell configuration

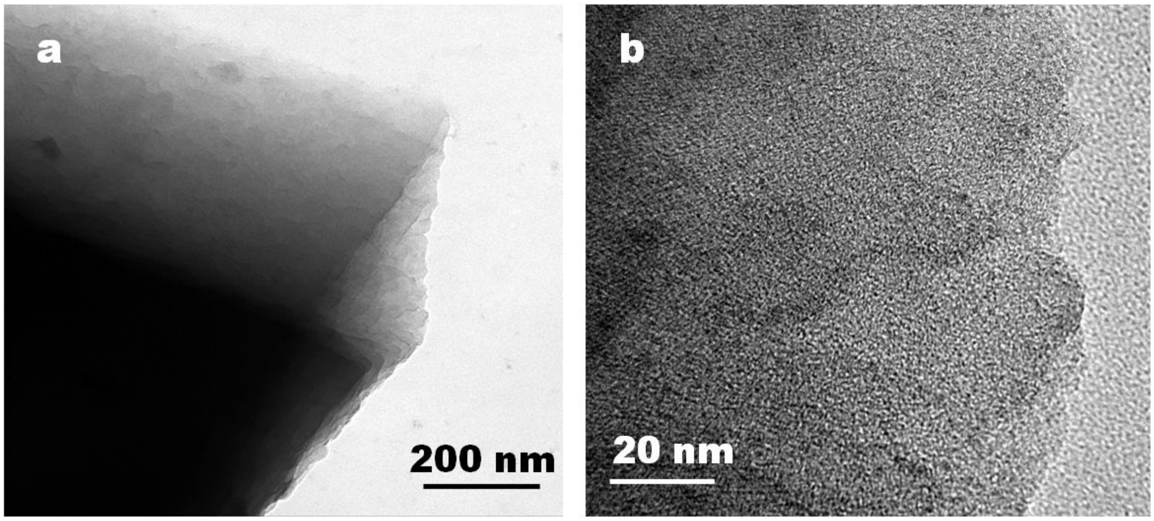

Figure S6. (a) Low-magnification TEM image of PAC-800. (b) High-magnification TEM image of PAC-800

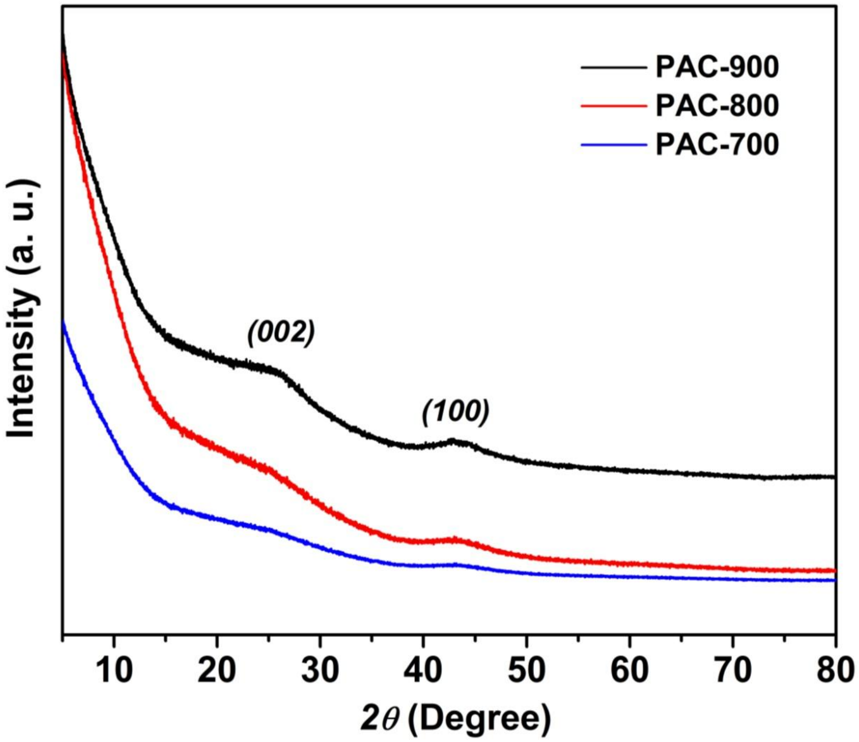

Figure S7. XRD patterns of PAC samples

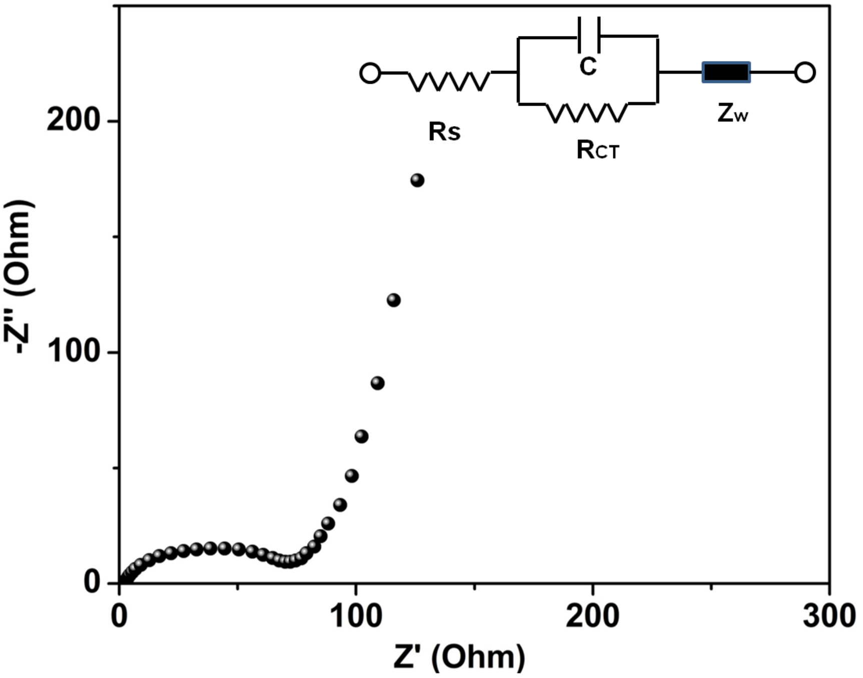

Figure S8. Nyquist plots and fitting circuit of Sn-C//PAC device

Table S1. Physicochemical of PACs.

Samples	S_{BET} (m^2g^{-1})	V_t (cm^3g^{-1})	***XPS analysis*** (atom%)		
			C	***N***	***O***
PAC-700	1064	0.58	58.86	0.45	33.69
PAC-800	1525	0.80	88.75	0	11.25
PAC-900	2167	0.98	98.23	0	1.77

Note: S_{BET}: specific surface area, V_t: pore volume.

Table S2. A comparasion of reported LIC systems with our Sn-C//PAC configuration.

LIC systems	*Energy density and corresponding power density*	*Cycling stability*	*Ref.*
NAC-400-Si/C	230 Wh kg^{-1}@ 1747 W kg^{-1}<br>141 Wh kg^{-1}@ 30127 W kg^{-1}	76.3% after 8000 cycles	11
B-Si/SiO_2/C//PSC	128 Wh kg^{-1}@1229 W kg^{-1}<br>89 Wh kg^{-1}@ 9704 W kg^{-1}	70% after 6000 cycles	5
TiC//PHPNC	101.5 Wh kg^{-1}@ 450 W kg^{-1}<br>23.4 Wh kg^{-1} @ 67500 W kg^{-1}	83% after 5000 cycles	12
3D-MnO/CNS//CNS	184 Wh kg^{-1}@ 83 W kg^{-1}<br>90 Wh kg^{-1}@15000 W kg^{-1}	76% after 5000 cycles	42
C//SnO_2–C	110 Wh kg^{-1}@ 190 W kg^{-1}<br>45 Wh kg^{-1}@ 2960 W kg^{-1}	80% after 5000 cycles	20
TiO_2-rGO//AC	42 Wh kg^{-1}@800 W kg^{-1}<br>8.9 Wh kg^{-1} @8000 W kg^{-1}	80% after100 cycles	43
F-Fe_2O_3//AC	28 Wh kg^{-1} @ 550 W kg^{-1}	90% after15000 cycles	44
Fe_3O_4/G//graphene	147 Wh kg^{-1}@ 150 W kg^{-1}<br>86 Wh kg–1@2587 W kg^{-1}	70%, 1000 cycles	19
Graphene/$Li_4Ti_5O_{12}$ //Graphene	95 Wh kg^{-1}@ 45 W kg^{-1}<br>32 Wh kg–1@ 3000 W kg^{-1}	87%, 500 cycles	45
CNT/V_2O_5//AC	25.5 Wh kg^{-1}@ 40 W kg^{-1}<br>6.9 Wh kg–1@6300 W kg^{-1}	80%, 10000 cycles	46
TiO_2 belt//Graphene	82 Wh kg^{-1} @ 570 W kg^{-1}	73%, 600 cycles	47

	21 Wh kg–1@19000 W kg^{-1}		
Patterned Si//AC	90 Wh kg^{-1}@ 90 W kg^{-1}<br>55 Wh kg–1@ 900 W kg^{-1}	100%,15000 ycles	48
$LiTi_2(PO_4)_3$//AC	14 Wh kg^{-1}@ 180 W kg^{-1}	~68%, 250 cycles	49
Nb_2O_5@Carbon //MSP-20	63 Wh kg^{-1} @ 70 W kg^{-1}<br>5 Wh kg^{-1}@16500 W kg^{-1}	100%, 1000 cycles	50
Ti doped Nb_2O_5//PANI-CNT	110 Wh kg^{-1} @ 150 kW kg^{-1}<br>23.6 Wh kg^{-1}@ 3000 W kg^{-1}	71 %, 2000 cycles	51
Graphene-VN //carbon nanorods	162 Wh kg^{-1}@ 200 W kg^{-1}<br>64 Wh kg^{-1}@10000 W kg^{-1}	86%, 1000 cycles	52
AC//soft carbon	48 Wh kg^{-1}@ 9000 W kg^{-1}	~	23
$H_2Ti_6O_{13}$//CMK-3	90 Wh kg^{-1}@ 11000 W kg^{-1}	80%, 1000 cycles	53
T-Nb_2O_5/Graphene paper//AC	47 Wh kg^{-1} @ 393 W kg^{-1}<br>15 Wh kg^{-1}@18000 W kg^{-1}	93%, 2000 cycles	54
TiO_2 nanotube//OMC	25 Wh kg^{-1}@3000 W kg^{-1}	~	55
Graphite//URGO	106 Wh kg^{-1}@84 W kg^{-1}<br>85 Wh kg^{-1}@4200 W kg^{-1}	100% after 1000 cycles	56
LTO–AC//AC	90 Wh kg^{-1}@50 W kg^{-1}<br>32 Wh kg^{-1}@6000 W kg^{-1}	~	57
Sn-C//PAC	195.7 Wh kg^{-1}@ 731 W kg^{-1}<br>84.6 Wh kg^{-1}@ 24375 W kg^{-1}	70% after 5000 cycles	Our work
